# How intra-source imbalanced datasets impact the performance of deep learning for COVID-19 diagnosis using chest X-ray images

**DOI:** 10.1038/s41598-023-45368-w

**Published:** 2023-11-03

**Authors:** Zhang Zhang, Xiaoyong Zhang, Kei Ichiji, Ivo Bukovský, Noriyasu Homma

**Affiliations:** 1 Graduate School of Biomedical Engineering, Tohoku University, Sendai, 980-8576 Japan; 2 Department of General Engineering, National Institute of Technology, Sendai College, Sendai, 989-3128 Japan; 3 Institute of Development, Aging and Cancer, Tohoku University, Sendai, 980-8576 Japan; 4 Tohoku University Graduate School of Medicine, Tohoku University, Sendai, 980-8576 Japan; 5 Department of Computer Science, Faculty of Science, University of South Bohemia in Ceske Budejovice, 370 05 Ceske Budejovice, Czech Republic

**Keywords:** Machine learning, Image processing

## Abstract

Over the past decade, the use of deep learning has been widely increasing in the medical image diagnosis field. Deep learning-based methods’ (DLMs) performance strongly relies on training data. Therefore, researchers often focus on collecting as much data as possible from different medical facilities or developing approaches to avoid the impact of inter-category imbalance (ICI), which means a difference in data quantity among categories. However, due to the ICI within each medical facility, medical data are often isolated and acquired in different settings among medical facilities, known as the issue of intra-source imbalance (ISI) characteristic. This imbalance also impacts the performance of DLMs but receives negligible attention. In this study, we study the impact of the ISI on DLMs by comparison of the version of a deep learning model that was trained separately by an intra-source imbalanced chest X-ray (CXR) dataset and an intra-source balanced CXR dataset for COVID-19 diagnosis. The finding is that using the intra-source imbalanced dataset causes a serious training bias, although the dataset has a good inter-category balance. In contrast, the deep learning model performed a reliable diagnosis when trained on the intra-source balanced dataset. Therefore, our study reports clear evidence that the intra-source balance is vital for training data to minimize the risk of poor performance of DLMs.

## Introduction

The severe acute respiratory syndrome Coronavirus 2 (SARS-CoV-2) causes the Coronavirus disease 2019 (COVID-19) that has been widely spread worldwide and continues to have a devastating effect on the health and life of the global population^1^. The polymerase chain reaction (PCR) test is the gold standard^2^ for detecting SARS-CoV-2 nowadays. Nevertheless, PCR testing is time-consuming and laborious, and it is also suffering from the high cost^3^.

As one of the essential complements to PCR testing, chest X-ray (CXR) imaging has also demonstrated its effectiveness in current diagnosis^4^. The CXR imaging is often part of the standard procedure for patients with respiratory complaints, and it is reported that some patients showed abnormalities in the CXR images before they eventually test positive for COVID-19 with the PCR test^4^. Moreover, all the rapid triaging, availability, accessibility, and portability of CXR imaging indicated that it could be a preliminary tool for COVID-19 screening. Nonetheless, one of the biggest bottlenecks of CXR screening is the need for experts to diagnose from the CXR images because the radiological signatures can be subtle.

The deep learning-based methods (DLMs) can actually enhance the diagnosis performance by radiologists^5^ and they can aid image diagnosis in the lung areas that is not easy even for the experts^6^. The success made by DLMs encouraged researchers to develop deep learning-based computer-aided diagnostic (CAD) systems that can aid radiologists in screening COVID-19 more rapidly and accurately^7–11^. Brunese et al.^7^ applied VGG-16 model for COVID-19 detection from CXR images. Hemdan et al.^8^ proposed an original COVIDX-Net framework to assist radiologists to automatically diagnose COVID-19 in CXR images. Kundu et al.^9^ proposed an ET-NET for a more sensitive computer tomography scan based COVID-19 detection. Saha et al.^10^ proposed a GraphCovidNet to deal with classification of COVID-19 or any other kind of pneumonia patients from healthy people in screening. Wang et al.^11^ also proposed the new architecture of deep learning model named COVID-Net for COVID-19 detection in CXR images. In these previous studies, the deep learning models could achieve high performance on COVID-19 detection.

Although the deep learning models can achieve high performance on COVID-19 detection, the lack of accepted theoretical explanation remains the fundamental problem of deep learning, i.e., the black-box problem^12^. The cause is that deep learning models lack transparency and explainability; it is difficult to know and understand how the model made a prediction, and the inner workings remain opaque to the outside observer^13^. Without a sufficient understanding of the machine-made prediction, it becomes very complicated to detect errors in models’ performance^13^, i.e., training bias caused by mislabeled training data, especially for medical applications. Therefore, the reliability of deep learning models remains a concern.

**Figure 1:**
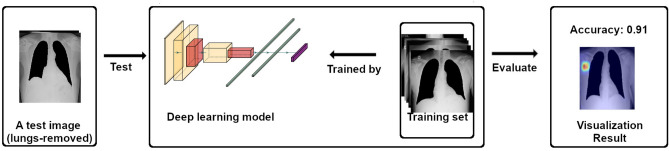
An overview of the previous study^14^. CXR images with lung regions removed are utilized to investigate the reliability of deep learning models for COVID-19 classification. Deep learning models can achieve high accuracy when images with lung regions are removed, and the focused locations are outside the lung regions when deep learning models make a COVID-19 prediction. The result indicates the deep learning models are unreliable in terms of medical findings, but the cause of the unreliable performance is still unknown.

For assessing the reliability of deep learning models used for COVID-19 detection in CXR images, Sadre et al.^14^ proposed a region-of-interest (ROI) hide-and-seek protocol. As shown in Fig. 1, to observe the reliability of these deep learning models, they removed lung regions from CXR images in a public CXR dataset and used them to train and test deep learning models. Then, a gradient-weighted class activation mapping (Grad-CAM) method^15^ was utilized to visualize which parts of the CXR images were focused on by the deep learning models. The experiment results showed that the deep learning models even could achieve high performance using the lungs-removed images, and the focused locations were outside the lung regions when deep learning models made a COVID-19 prediction. Results in this study^14^ indicated the deep learning models are unreliable in terms of medical findings because the image features contributing to COVID-19 classification exist outside the lung regions, which is unexpected for a lung-based illness^14^.

The study^14^ mentioned that the unreliability of DLMs might be explained via data characteristics, because the previous studies collected as much data as possible from different medical facilities to develop DLMs for the urgent pandemic. The inter-category imbalance (ICI), i.e., the difference in data quantity among categories, belongs among such data characteristics, and its impact on DLMs has attracted much attention from researchers^16,17^. At the same time, there are few investigations for intra-source imbalance (ISI), which means the ICI within the data collected from each medical facility. Therefore, to demonstrate the unreliable performance shown in the previous study^14^ is related to the ISI, we organized two different COVID-19 datasets and analyzed how the ISI affects DLMs’ performance. The both datasets consist of positive and negative categories, and they are well-balanced between the two categories. The data sources differ between the two datasets (see Table 1). One dataset (Qata-COV19) was collected from different medical facilities, and every single facility only provided positive or negative images. As one of the largest open-access COVID-19 dataset, the Qata-COV19 dataset has been used to train and test deep learning models in many previous studies^18,19^. In another dataset (BIMCV), positive and negative CXR images were collected from a single medical facility. The ROI hide-and-seek protocol was implemented on the two datasets to investigate the effect of the ISI on the deep learning models. Then, to evaluate the reliability of the deep learning models trained by each dataset, we made a cross-dataset test, which refers to training a deep learning model on one dataset and testing it on another dataset. Finally, we analyzed the relationship between the unreliability and the ISI according to the experimental results.

The outline of this paper is as follows. Firstly, we discuss the unreliability of deep learning models in terms of medical findings, as shown in the previous study^14^. Then, we introduce the materials and methods for clarifying the relationship between the unreliability and the ISI. Finally, we summarized and analyzed the experimental results.

## Materials and methods

### Datasets

**Figure 2:**
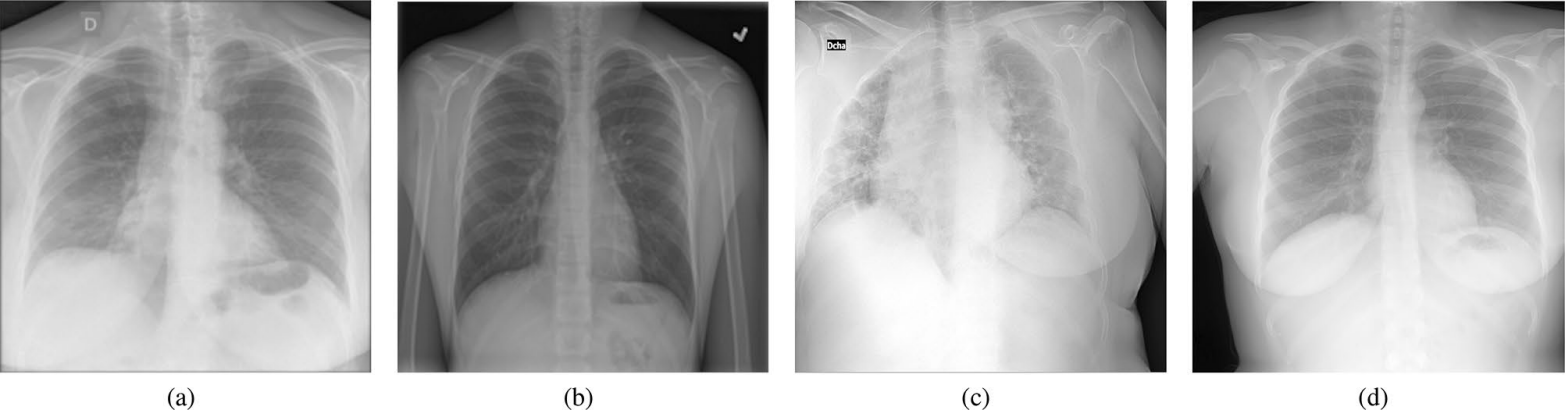
Examples of positive and negative CXR images in the two datasets: (**a**) a positive CXR image in the Qata-COV19 dataset, (**b**) a negative CXR image in the Qata-COV19 dataset, (**c**) a positive CXR image in the BIMCV dataset, (d) a negative CXR image in the BIMCV dataset.

In this study, we used two CXR datasets collected from various public COVID-19 databases to investigate how the ISI of training data impacts the deep learning models for the COVID-19 diagnosis. The intra-source imbalanced dataset is Qata-COV19 dataset^20^, and the intra-source balanced dataset is BIMCV dataset^21,22^. As shown in Table 1, the Qata-COV19 dataset contains 3761 positive CXR images from five different public facilities and 3761 negative CXR images from seven other public facilities. In comparison, the BIMCV dataset contains 2461 positive CXR images and 2461 negative CXR images from a single public facility, Valencian Region Medical ImageBank. In the Qata-COV19 dataset, one facility only provided CXR images in a single category. For example, BIMCV+^21^ only provided positive images, and RSNA dataset^23^ only provided negative images for the Qata-COV19 dataset. The two datasets are both well balanced between positive and negative to avoid the influence of ICI. Since Qata-COV19 contains positive images not only from BIMCV but also from other medical facilities, the two dataset have different sizes. Examples of positive and negative images in each dataset are shown in Fig. 2. The important relationship between the Qata-COV19 dataset and the BIMCV dataset is that both shared the positive CXR images from BIMCV+ but did not share any negative CXR images.

**Table 1:** Our study used two image datasets (Qata-COV19, BIMCV); Qata-COV19 has images provided from various facilities and only for a single category, while BIMCV collected images from the same facility.

Dataset	Category	Data Source	Train	Test
Qata-COV19^20^	Positive	BIMCV+^21^	3383	378
		MHH^24^		
		SIRM^25^		
		COVID-chestxray dataset^26^		
		COVID-19 radiography dataset^27^		
	Negative	RSNA^23^	3383	378
		Padchest dataset^28^		
		Guangzhou Women’s Medical Center^29^		
		Indiana Network for Patient Care^30^		
		MC dataset^31^		
		Shenzhen Hospital^31^		
		ChestX-ray14 dataset^32^		
BIMCV	Positive	BIMCV+^21^	2222	239
	Negative	BIMCV-^22^	2222	239

In our study, the two datasets were used to clarify the influence of the ISI on the reliability of deep learning models. All the images were resized to $512 \times 512$ pixels. The datasets were divided into training and testing subsets according to Table 1.

**Figure 3:**
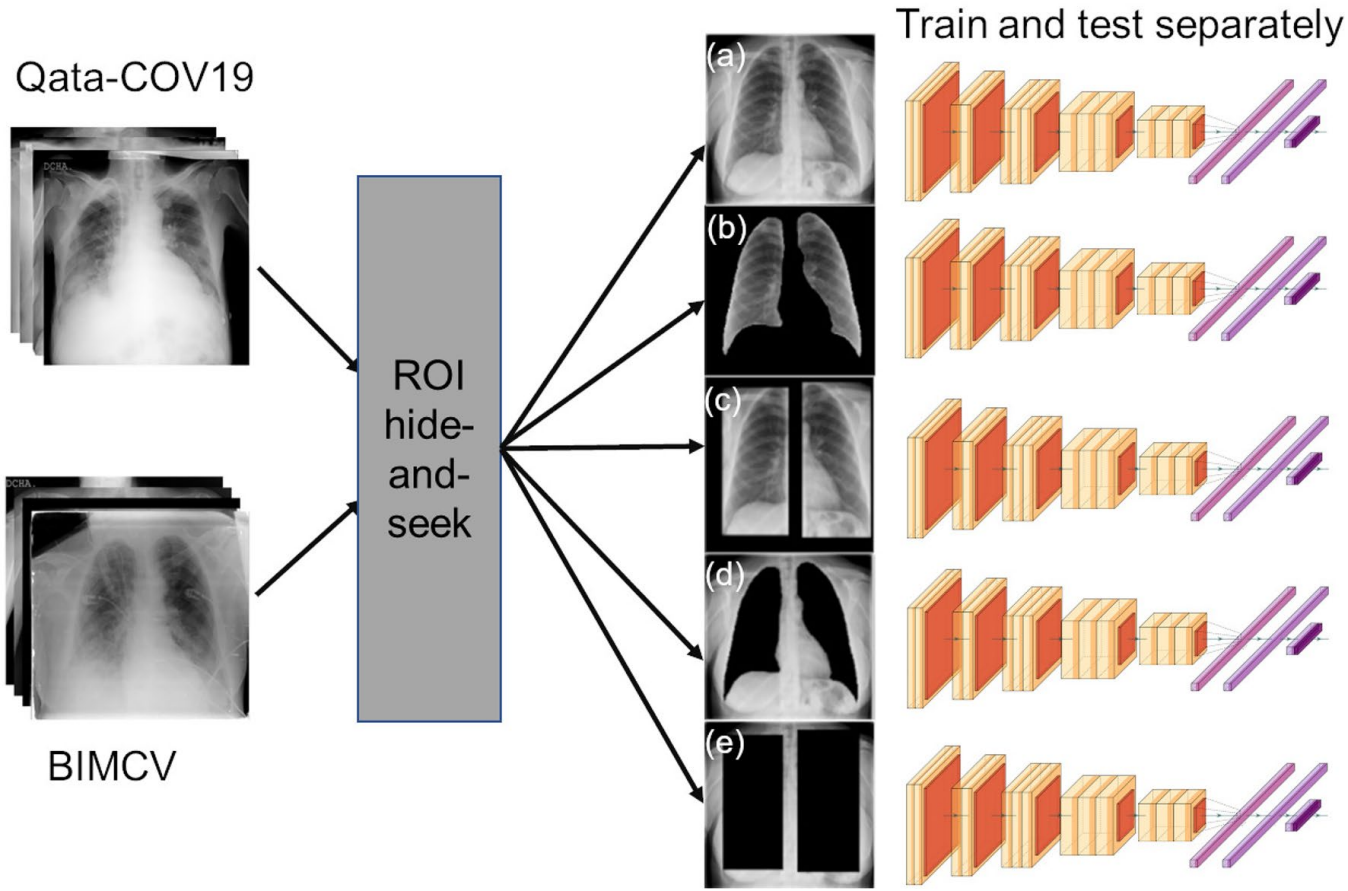
Overview of the comparative experiment. ROI hide-and-seek protocol operated (**a**) original images from the Qata-COV19 dataset or the BIMCV dataset to emphasize and hide the lung regions, respectively. (**b**) lungs-isolated images and (**c**) lungs-framed images were generated by emphasizing the lung regions, while (**d**) lungs-removed images and (**e**) lungs-boxed-out images were generated by hiding the lung regions. The original datasets and the modified datasets were utilized to train and test a VGG-16 model separately.

### Experiments

As in Fig. 3, to clarify the relationship between the ISI of training data and the reliability of deep learning models, we re-implemented the ROI hide-and-seek protocol^14^ on the Qata-COV19 dataset and the BIMCV dataset and trained and tested the VGG-16 model^33^ on the original datasets and the modified datasets separately.

At first, we re-implemented the ROI hide-and-seek protocol^14^ to generate datasets. In this step, we used a pre-trained U-Net model^14^ to segment the lung regions from the original images (Fig. 3a). According to the segmented lung regions, bounding boxes around the lungs were also generated. Four types of modified images were generated by emphasizing and hiding the lung regions and the bounding boxes. Lungs-isolated images (Fig. 3b) and lungs-framed images (Fig. 3c) were generated by isolating the segmented lung regions and regions inside the bounding boxes from the original images, respectively; lungs-removed images (Fig. 3d) and lungs-boxed-out images (Fig. 3e) were generated by removing the segmented lung regions and regions inside the bounding boxes from the original images, respectively. We can see that original images, lungs-isolated images, and lungs-framed images are all with lung regions, while lungs-removed images are without lung regions. Lungs-boxed-out images are without lung regions or lung borders.

A VGG-16 model pre-trained with ImageNet was used in this study. We used a global average pooling layer to replace the first fully-connected layer. The VGG-16 model was trained to classify the original images or modified CXR images into positive and negative classes. We tuned all the weights and biases in the VGG-16 model during the training step.

In the first experiment, we trained VGG-16 models by using original images and four types of modified images from the Qata-COV19 dataset separately to investigate the effect of lung regions on the performance of the VGG-16 model. And for investigating the effect when using an intra-source balanced dataset, we trained VGG-16 models by using original images and four types of modified images from the BIMCV dataset, separately.

In addition, a cross-dataset test^34,35^ was used to evaluate the reliability of the models trained by different datasets. We trained a VGG-16 model using the original images from one dataset and then tested them on the authentic images from another dataset.

To evaluate the performance of the deep learning models, we utilized a receiver operating characteristics (ROC) curve^36^ and the Area Under ROC curve (AUC).

## Results

**Figure 4:**
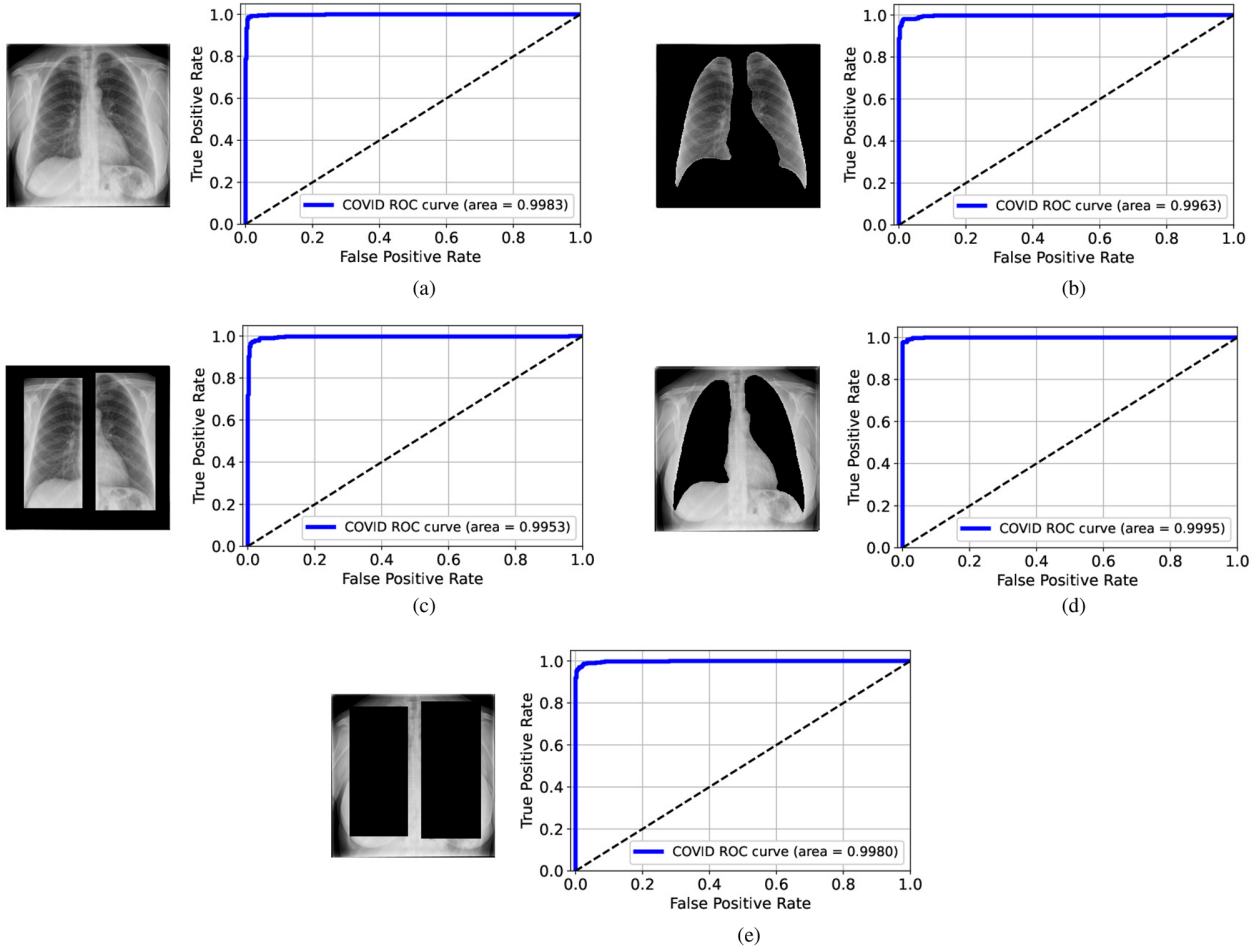
ROC curves for the VGG-16 models trained and tested on the modified datasets from Qata-COV19: (**a**) original images, (**b**) lungs-isolated images, (**c**) lungs-framed images, (**d**) lungs-removed images, and (**e**) lungs-boxed-out images. The deep learning models achieved high performance, even with hidden lung regions.

**Figure 5:**
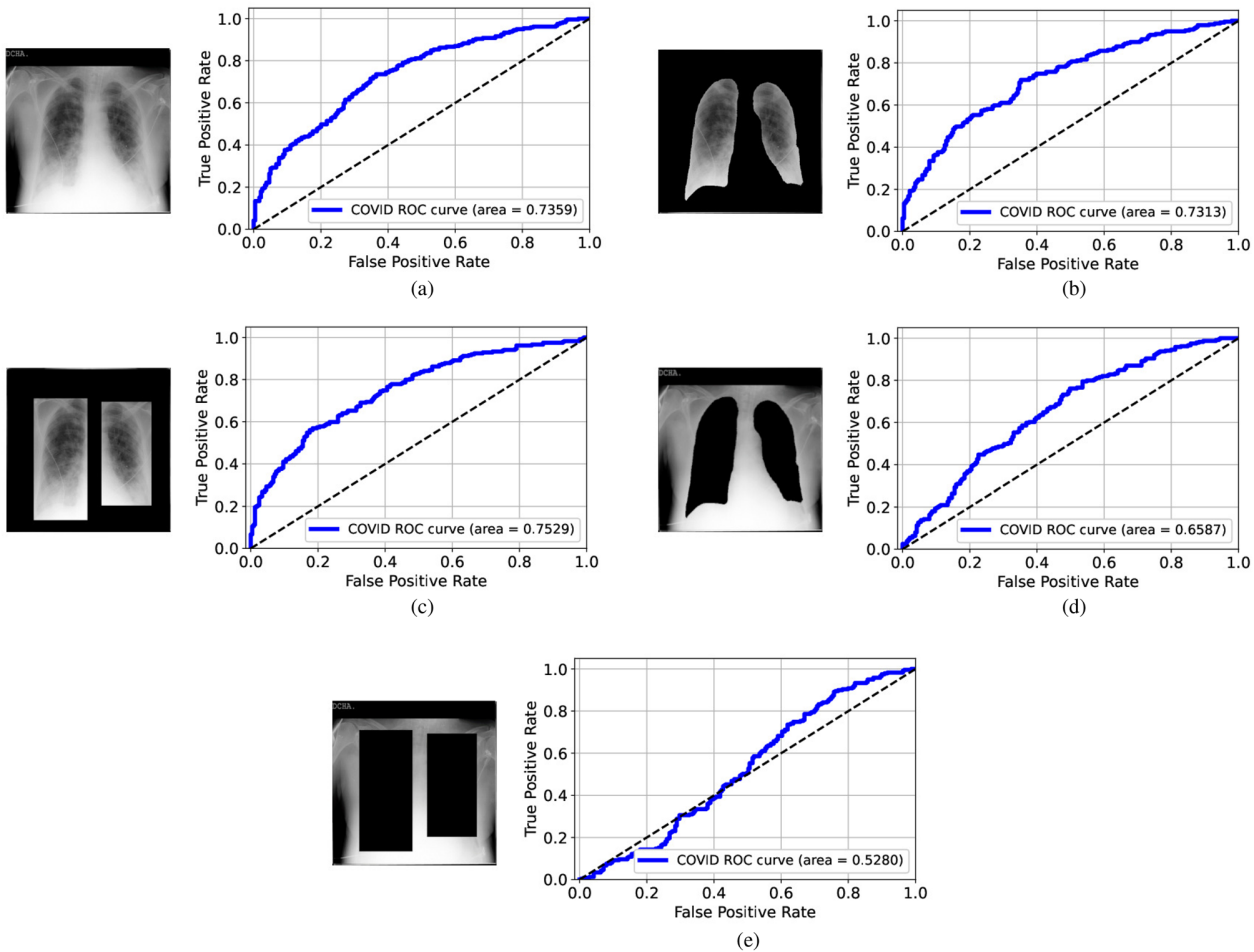
ROC curves for the models trained and tested on the modified datasets from BIMCV: (**a**) original images, (**b**) lungs-isolated images, (**c**) lungs-framed images, (**d**) lungs-removed images, and (**e**) lungs-boxed-out images. The performance degraded a lot when lung regions were hidden.

**Figure 6:**
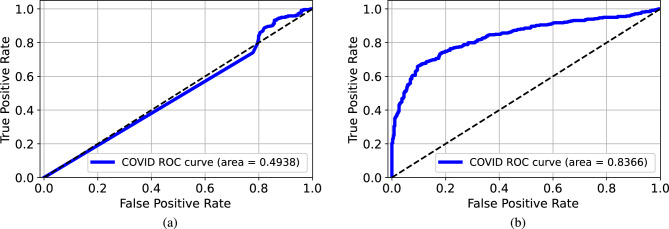
ROC curves for the cross-dataset test: (**a**) testing the Qata-COV19-trained model on the BIMCV dataset, (**b**) testing the BIMCV-trained model on the Qata-COV19 dataset. The model trained on original images from BIMCV dataset was able to classify original images from the Qata-COV19 dataset, while the model trained on original images from the Qata-COV19 dataset failed to classify original images from the BIMCV dataset.

As in Fig. 4, the AUC values were all larger than 0.99 when the VGG-16 model was trained and tested on the original images or modified images from Qata-COV19. According to the ROC curves, the VGG-16 model achieved relatively high performance even when lung areas were removed or boxed out, which showed the same results as in the previous study^14^. These results confirm the high risk of obtaining an unreliable deep learning model. As shown in Fig. 5, when using the lungs-removed images or lungs-boxed-out images from BIMCV, the AUC values degraded a lot. The results showed that image features inside the lung regions played a more important role in classification using an intra-source balanced dataset. Such different results with different datasets demonstrate that the unreliable performance is related to the ISI.

As shown in Fig. 6a, when testing the BIMCV-trained model on the original CXR images from the Qata-COV19 dataset, the AUC was nearly 0.5, and the performance was the same as a random classifier. The ROC curve shows that the model failed to classify the positive and negative images from BIMCV. The result demonstrates lacking balance in data sources leads to unreliability. On the other hand, as shown in Fig. 6b, when testing the Qata-COV19-trained model on the original CXR images from the BIMCV dataset, the AUC was 0.8863, and the model trained by BIMCV was able to classify positive and negative CXR images in the Qata-COV19 dataset. Moreover, when testing the Qata-COV19-trained model on BIMCV dataset, the specificity was 0, which showed that all the images from the BIMCV dataset were classified into the positive class even if they were negative.

## Discussion

### Cross-validation

To demonstrate the statistical significance of the experiments, we utilized cross-validation^37^, which uses different portions of the data to train and test a model on different iterations, in the comparison experiments and the cross-dataset test. Cross-validation is a statistical technique for testing the performance of deep learning models that can help to avoid selecting bias.

**Figure 7:**
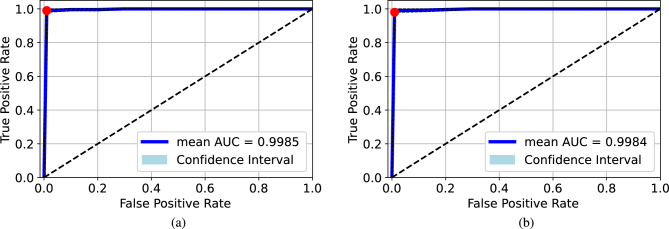
Mean cross-validated ROC curves and 95% confidence intervals for the cross-validation in Qata-COV19 dataset: (**a**) original images, (**b**) lungs-boxed-out images. Absence of the lung regions did not significantly affect the performance.

**Figure 8:**
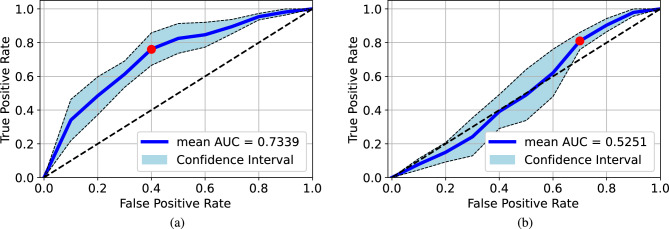
Mean cross-validated ROC curves and 95% confidence intervals for the cross-validation in BIMCV dataset: (**a**) original images, (**b**) lungs-boxed-out images. Absence of lung regions significantly impacted the deep learning performance. Red points are the cut-off points. Red points are the cut-off points.

To demonstrate the effect of lung regions on the deep learning performance, we ran a 5-folder cross-validation to compare the impact of lung regions when using Qata-COV19 dataset and BIMCV dataset. In the cross-validation, original images and lungs-boxed-out images were used to train and test a VGG-16 model separately. We compared the mean cross-validated ROC curves and 95% confidence intervals. As shown in Fig. 7, the models achieved $0.9983 \pm 0.0015$ and $0.9984 \pm 0.0013$ AUC values for the original images and the lungs-boxed-out images, respectively. Absence of the lung regions did not significantly affect the performance when using Qata-COV19 for training and test. On the other hand, as shown in Fig. 8, when using BIMCV dataset, the model trained by lung-boxed-out images performed worse than the model trained by original images. The model trained by original images achieved $0.7339 \pm 0.0454$ AUC value and the model trained by lungs-boxed-out images achieved $0.5250 \pm 0.0751$ AUC value. Absence of lung regions significantly impacted the deep learning performance when using BIMCV dataset. Moreover, we found out the optimal cut-off points^38^ of the ROC curves by maximizing sensitivity (True Positive Rate) plus specificity (True Negative Rate). The model trained by the original images and the lungs-boxed-out images from Qata-COV19 dataset both achieved more than 0.99 accuracy on the cut-off points. On the other hand, the model trained by the original images and the lungs-boxed-out images from BIMCV dataset achieved 0.68 and 0.55 accuracy on the cut-off points, respectively.

**Figure 9:**
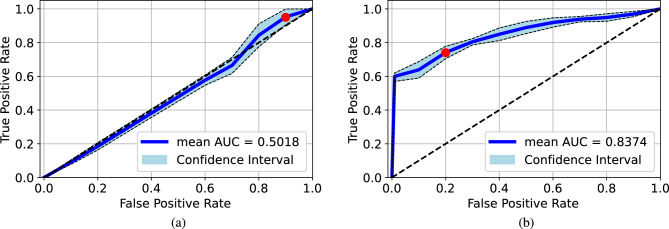
Mean cross-validated ROC curves and 95% confidence intervals for the cross-validation in the cross-dataset test: (**a**) testing the Qata-COV19-trained model on the BIMCV dataset, (**b**) testing the BIMCV-trained model on the Qata-COV19 dataset. The model trained by BIMCV performed more reliable than the model trained by Qata-COV19 dataset. Red points are the cut-off points.

We also ran a 5-folder cross-validation for the cross-dataset test by using the original images from BIMCV and Qata-COV19 datasets. Based on the results of the cross-validation, the ROC curves and the 95% confidence intervals are given in Fig. 9. As a result, the model trained by the Qata-COV19 achieved $0.5018 \pm 0.0171$ AUC value on the BIMCV dataset and the model trained by the BIMCV achieved $0.8374 \pm 0.0158$ AUC value on the Qata-COV19 dataset. As for the accuracy, the model trained by images from Qata-COV19 and BIMCV achieved 0.51 and 0.76 accuracy on the cut-off point, respectively. The result significantly demonstrated the model trained by the BIMCV dataset performed more reliable than the model trained by the Qata-COV19 dataset.

### Visualization

To provide intuitive explanations for the unreliable performance of the model trained on the Qata-COV19 dataset, we utilized Local Interpretable Model-agnostic Explanations (LIME)^39^ method to visualize the basis of the predictions made by the VGG-16 models in the cross-dataset test. The LIME method can generate a readily interpretable model which is locally close to the deep learning model and highlight areas inside input images that contribute to predictions. We selected top-5 areas which contribute the most in the LIME results as the explanations for predictions.

**Figure 10:**
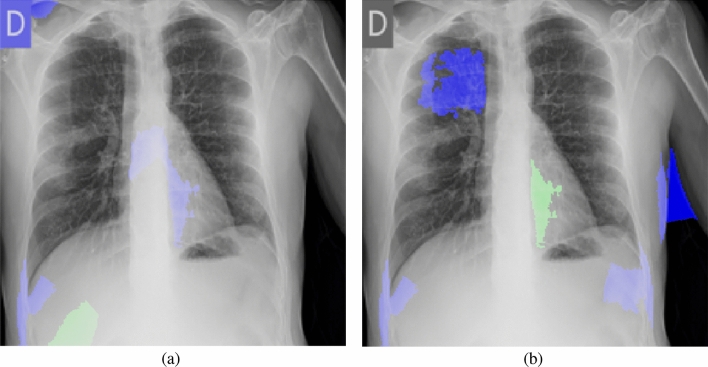
The LIME explanations for classifying a positive case from BIMCV dataset. (**a**) The model trained by the Qata-COV19 dataset focused on the marker and classified it into positive class. (**b**)The model trained by the BIMCV dataset focused inside lung regions and classified it into positive class. Blue areas contributed positively to the predictions and green areas contributed negatively to the predictions.

**Figure 11:**
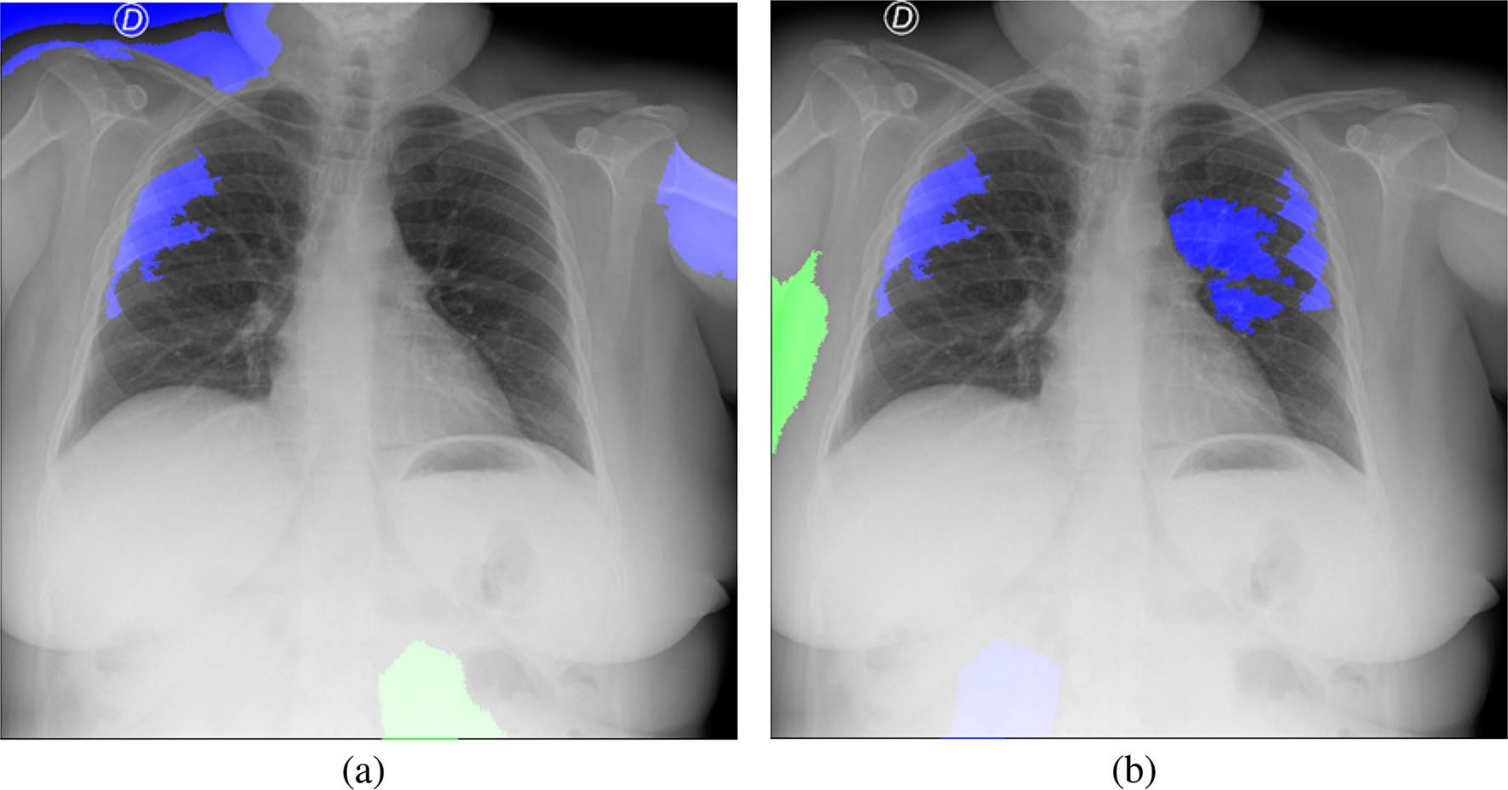
The LIME explanations for classifying a negative case from BIMCV dataset. (**a**) The model trained by the Qata-COV19 dataset focused on the marker and classified it into positive class. (**b**)The model trained by the BIMCV dataset focused inside lung regions and classified it into negative class. Blue areas contributed positively to the predictions and green areas contributed negatively to the predictions.

Figure 10 shows the LIME explanations for classifying a positive case from BIMCV dataset. In this case, both of the models made a true prediction. As shown in Fig. 10a, the model trained by the Qata-COV19 dataset focused more on the marker and areas outside lung regions. In contrast, as shown in Fig. 10b, the model trained by the BIMCV dataset focused more inside the lung regions. Figure 11 shows the LIME explanations for a negative case from BIMCV dataset. The model trained by BIMCV made a true prediction but the model trained by Qata-COV19 dataset made a false prediction. As shown in Fig. 11a, the model trained by the Qata-COV19 dataset focused on the marker and areas outside lung regions and classified this negative image into positive class. Since the markers represented the BIMCV data source, the results demonstrated the model trained by the Qata-COV19 dataset learned the features representing data sources but not the features representing COVID-19 characteristics. The visualization results showed the features representing data sources can strongly impact the decisions of the model trained by intra-source imbalanced dataset.

Our study reveals that the ISI of training data can lead to an unreliable performance of deep learning models. The analysis of the comparative experiment and the cross-dataset test are as follows:

- The VGG-16 model performed well even when lung regions were hidden when using the Qata-COV19 dataset. This result shows the same unreliable performance as shown in the previous study^14^.
- The performance degraded when lung regions were hidden from the CXR images when using the BIMCV dataset. In particular, the ROC curve suggested nearly no capacity for classification when lung regions were boxed out. It demonstrated that the classification of CXR images in the BIMCV dataset relies on the features representing COVID-19 characteristics in lung regions, and the deep learning models are more reliable when using intra-source balanced datasets.
- The model trained by the Qata-COV19 dataset showed nearly no capacity to classify the CXR images in the BIMCV dataset because the AUC value was about 0.5. Moreover, according to the sensitivity and specificity, all images from BIMCV were classified into the COVID-19 positive class. It is suggested that the model learned the features representing data characteristics of BIMCV from the positive images in the training step, so that the negative images from the BIMCV dataset were also classified into positive class in the test step. It revealed that when using intra-source imbalanced datasets, the prediction bases are the features representing each data source characteristics, but not the features representing COVID-19 characteristics, so the ISI can lead to an unreliable performance of deep learning models. Especially, as shown in the cross-dataset test, the model trained by intra-source imbalanced datasets can be totally unable to make a diagnosis for other datasets.
- The model trained by the BIMCV dataset achieved a relatively high performance when testing on the Qata-COV19 dataset, which indicated it had better generalizability.

Many previous studies^18,19^ used the Qata-COV19 dataset to train and test deep learning models and obtained high performance on the test subset, but few of them discussed about the reliability and generalizability. Our study revealed a risk of training bias when using such an intra-source imbalanced dataset, so researchers should raise their concerns about the intra-source balance when collecting training data to minimize the risk of unreliability.

## Conclusion

We report that the intra-source imbalance of training data leads to the unreliability of deep learning methods by re-implementing the ROI hide-and-seek protocol on two differently collected CXR datasets. Using a cross-dataset test, we show that the model trained by intra-source imbalanced datasets might classify images based on the features characterizing data sources; hence, it lacks the capability to diagnose other datasets. As emphasized in the introduction, for the urgent COVID-19 pandemic, many previous studies collected as much data as possible from different medical facilities to train deep networks, but without enough validation. They might lack clinical applicability because of the intra-source imbalance of the training data. Our study reveals the risk of unreliability when using intra-source imbalanced datasets in deep learning methods, not only for COVID-19 classification but also for other medical applications. Therefore, when developing deep learning methods, we should ensure the intra-source balance of the datasets before they are applied to train deep learning models.

## Data Availability

The datasets generated and/or analysed during the current study are available in the Kaggle repository and the IEEE dataport repository: https://www.kaggle.com/aysendegerli/qatacov19-dataset, https://dx.doi.org/10.21227/w3aw-rv39, and https://dx.doi.org/10.21227/m4j2-ap59.
